# Role of DNA-Dependent Protein Kinase in Mediating Cyst Growth in Autosomal Dominant Polycystic Kidney Disease

**DOI:** 10.3390/ijms221910512

**Published:** 2021-09-29

**Authors:** Ashley N. Chandra, Sayanthooran Saravanabavan, Gopala K. Rangan

**Affiliations:** 1 Michael Stern Laboratory for Polycystic Kidney Disease, Westmead Institute for Medical Research, The University of Sydney, Westmead, NSW 2145, Australia; ashley.chandra@sydney.edu.au (A.N.C.); sayan.saravanabavan@sydney.edu.au (S.S.); 2 Department of Renal Medicine, Westmead Hospital, Western Sydney Local Health District, Westmead, NSW 2145, Australia

**Keywords:** DNA-dependent protein kinase, DNA damage signaling, double strand break, kinase inhibitors, proliferation, kidney cyst

## Abstract

DNA-dependent protein kinase (DNA-PK) is a serine/threonine protein involved in DNA damage response (DDR) signaling that may mediate kidney cyst growth in autosomal dominant polycystic kidney disease (ADPKD) due to its pleiotropic effects on proliferation and survival. To test this hypothesis, the expression of DNA-PK in human ADPKD and the in vitro effects of DNA-PK inhibition in a three-dimensional model of Madin-Darby Canine Kidney (MDCK) cyst growth and human ADPKD cells were assessed. In human ADPKD, the mRNA expression for all three subunits of the DNA-PK complex was increased, and using immunohistochemistry, the catalytic subunit (DNA-PKcs) was detected in the cyst lining epithelia of human ADPKD, in a focal manner. In vitro, NU7441 (a DNA-PK kinase inhibitor) reduced MDCK cyst growth by up to 52% after long-term treatment over 6–12 days. Although human ADPKD cell lines (WT9-7/WT9-12) did not exhibit synthetic lethality in response to DNA-PK kinase inhibition compared to normal human kidney cells (HK-2), the combination of low-dose NU7441 enhanced the anti-proliferative effects of sirolimus in WT9-7 and WT9-12 cells by 17 ± 10% and 11 ± 7%, respectively. In conclusion, these preliminary data suggest that DNA-PK mediates kidney cyst growth in vivo without a synthetically lethal interaction, conferring cell-specificity in human ADPKD cells. NU7441 enhanced the anti-proliferative effects of rapamycin complex 1 inhibitors, but the effect was modest.

## 1. Introduction

Autosomal dominant polycystic kidney disease (ADPKD) is due to germ-line variants, predominantly in either PKD1 (85%) or PKD2 (15%), encoding the polytopic integral membrane, polycystin-1, and the calcium transient receptor channel, polycystin-2, respectively [1,2,3]. Both genotype as well as the total dose of ADPKD-causative genes, govern the total kidney cyst burden and severity of kidney disease [4,5,6]. The acquisition of somatic variants in the unaffected PKD allele has been hypothesized to cause a further reduction in gene dose and explain the focal nature of kidney cyst formation [7,8]. In previous studies, the genomic instability and DNA damage were increased in human and experimental models of PKD [9,10,11,12], suggesting that the DNA damage response (DDR) pathway is a potential therapeutic target in ADPKD [13].

Multiple DDR proteins work together, often with redundancy, to maintain genomic fidelity, despite exposure to numerous endogenous and exogenous genotoxic insults (~105 lesions each day) [14]. Inefficient repair of lesions by mutated or overexpressed genes results in DNA double strand breaks (DSBs), chromosomal rearrangement, and cancerous transformation [14]. In previous studies, pharmacological (but not genomic) attenuation of ataxia-telangiectasia mutated (ATM) protein kinase attenuated the proliferation of cystic epithelial cells in ADPKD [15,16]. Despite the redundancy of the DDR pathways in normal health, cancer cells exhibit selective reliance on certain DDR kinases for DNA repair and exhibit ‘synthetic lethality’ in response to DDR inhibition, and this has been the focus of some tumor-selective anti-cancer treatments [15,16].

DNA-dependent protein kinase (DNA-PK) belongs to the phosphatidylinositol-3-kinase related kinase (PI3KK) family and functions by non-homologous end joining (NHEJ) of double-strand breaks [15,16]. It is a holoenzyme comprising three subunits: (i) Ku 70 (*XRCC5*) and (ii) Ku80 (*XRCC6*), which translocate along DNA, bind to double strand breaks and recruit (iii) the large catalytic subunit, DNA-PKcs (*PRKDC*). In addition, DNA-PK induces centrosome amplification during stalled replication [17] and ciliogenesis following genotoxic injury [18], and reduces mitochondrial biogenesis [19]; key cellular processes associated with ADPKD progression [20,21,22,23]. In this regard, previous studies showed that PIK-75, a dual inhibitor of phosphoinositide 3-kinase (PI3K) and DNA-PK, slowed cyst growth and integrity in vitro in a high-throughput screening of protein kinases [24]. Furthermore, inhibition of DNA-PK sensitized cells to inhibitors of the mammalian target of rapamycin complex 1 (mTORC1), which delays the progression of PKD in experimental models but has adverse effects at clinical doses [25,26,27,28,29].

Therefore, the aim of this preclinical study was to investigate the role of DNA-PK in the pathogenesis of ADPKD using observational and in vitro studies to determine whether it could be a potential target in future in vivo studies. The following specific hypotheses were evaluated: (i) the expression of DNA-PK is increased in human ADPKD; (ii) the pharmacological inhibition of DNA-PK reduces cyst growth in vitro; (iii) inhibition of DNA-PK is selectively toxic to ADPKD cells; and (iv) DNA-PK inhibition enhances the sensitivity of ADPKD cells to sub-therapeutic doses of mTORC1 inhibition.

## 2. Results

### 2.1. The Expression of DNA-PK Is Upregulated in Human ADPKD Transcriptome

As shown in Table 1, compared to normal kidney, the expression of genes encoding the subunits of the DNA-PK complex: DNA-PKcs (*PRKDC*), Ku70 (*XRCC5*), and Ku80 (*XRCC6*) were increased in human ADPKD transcriptome. *PRKDC* was upregulated by 2.12-fold in ADPKD tissue (95% CI (1.78, 2.53); *q* < 0.001), increasing from 1.41-fold in minimally cystic tissue (95% CI (1.13, 1.76); *q* < 0.05) to 3.18-fold in large cysts (95%CI (1.41, 4.47); *q* < 0.05). *XRCC5* and *XRCC6* were also increased by 1.79-fold (95% CI (1.68, 1.90); *q* < 0.01) and 1.65-fold (95% CI (1.53, 1.78); *q* < 0.001), respectively, in ADPKD tissue, and this was maintained in all cyst sizes (Table 1).

### 2.2. Focal Increase of DNA-PKcs in Cyst Lining Epithelial Cells of Human ADPKD

**Figure 1:**
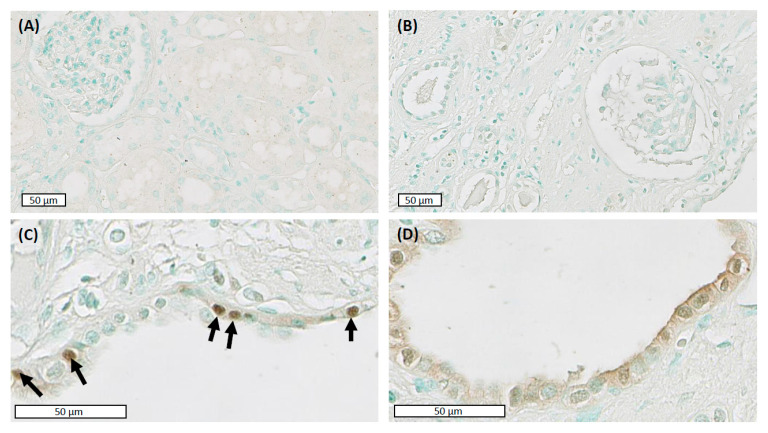
The catalytic subunit of DNA-dependent protein kinase (DNA-PKcs) is expressed focally in cyst lining epithelia. (**A**) Weak cytosolic expression in the normal renal cortex. (**B**) Non-cystic tissue in end-stage human autosomal dominant polycystic kidney disease (ADPKD) is negative for DNA-PKcs. (**C**) DNA-PKcs immunostaining of focal nuclei in cyst-lining epithelial cells (arrows). (**D**) Increased cytosolic and membranous staining in cyst lining epithelia in smaller cysts (100–200 µm). Representative photomicrographs taken at 40× magnification. Scale bars represent 50 µm.

In the normal kidney, immunostaining for DNA-PKcs was weak with cytosolic staining (with negative nuclei) in the proximal tubular epithelial cells (Figure 1A). In ADPKD, DNA-PKcs were absent in minimally cystic regions in either nuclei or cytosol (Figure 1B). In contrast, focal and random immuno-positive nuclei were detected in the cyst lining epithelia (Figure 1C). Stronger immunostaining for DNAPKcs was detected in the cytosol and apical membrane of epithelial cell lining dilated tubules and small cysts (50–200 µm) (Figure 1D). The specificity of staining for DNA-PKcs was confirmed on antibody-negative controls (data not shown).

### 2.3. Pharmacological Inhibition of DNA-PK MDCK Cyst Growth In Vitro

**Figure 2:**
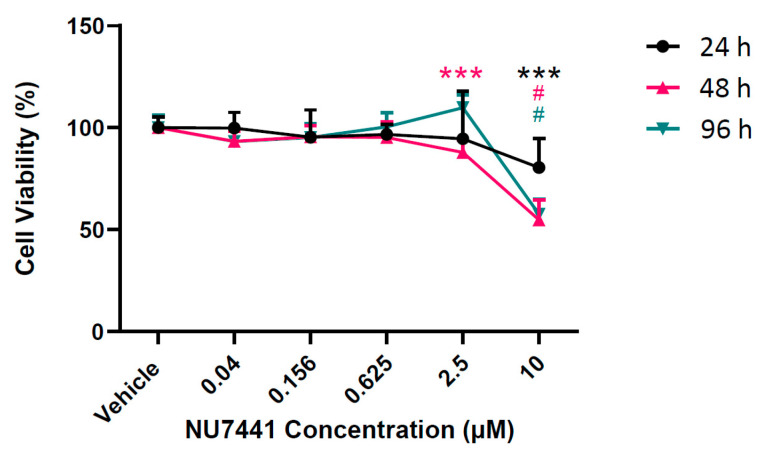
MTT assay assessing Madin-Darby canine kidney (MDCK) cell viability at 24, 48, and 96 h after exposure to NU7441 at various concentrations. Sample absorbances were normalized to vehicle control. Significance determined by non-parametric Kruskal–Wallis and Dunn–Bonferroni with control post hoc for MTT assay *** *p* < 0.001, # *p* < 0.0001 versus vehicle control at the same time point.

Before assessing the in vitro effects of NU7441 on MDCK cyst growth, the effect on the number of viable cells was first assessed using an MTT assay. As shown in Figure 2, the number of viable cells was reduced at all time points following NU7441 treatment at 10 µM, whereas lower doses were similar to the vehicle.

**Figure 3:**
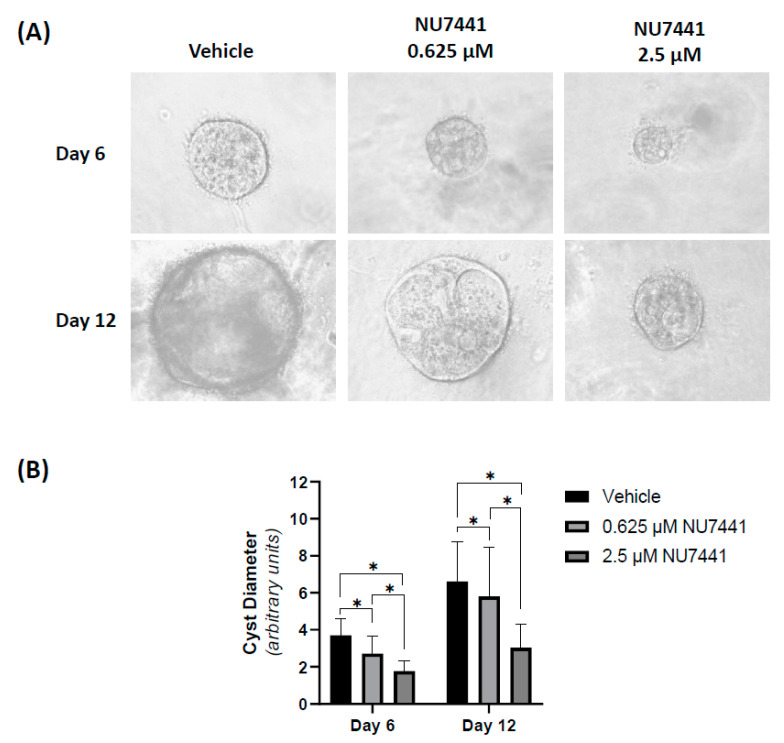
DNA-dependent protein kinase (DNA-PK) inhibitor NU7441 reduces cyst growth in a dose dependent manner. (**A**) Representative images of cysts (100× magnification) at days 6 and 12 with low (0.625 µM) and moderate (2.5 µM) doses of NU7441. (**B**) Cyst diameter measured at days 6 and 12 of prolonged exposure to NU7441, showing a dose-dependent decrease in cyst diameter. Results are presented as mean ± standard deviation (*n* = 180 for vehicle and 0.625 µM, and *n* = 120 for 2.5 µM NU7441 for each time point). Significance was determined by one-way analysis of variance (ANOVA) and post hoc Tukey honestly significant difference (HSD) test. * *p* < 0.01 versus vehicle control at the same time point.

Therefore, studies in MDCK cysts were performed using low (0.625 µM) and moderate (2.5 µM) doses. As shown in Figure 3, in forskolin-induced MDCK cysts, NU7441 reduced mean cyst diameter by 27% and 52% at 0.625 and 2.5 µM concentrations, respectively, on day 6 of treatment compared to the vehicle. This effect was sustained for 12 days of culture (Figure 3). No cyst formation when cells were treated with sirolimus (50 nM) or 10 µM NU7441 (data not shown).

### 2.4. DNA-PK Inhibition Does Not Cause Synthetic Lethality of Human ADPKD Cells 

**Figure 4:**
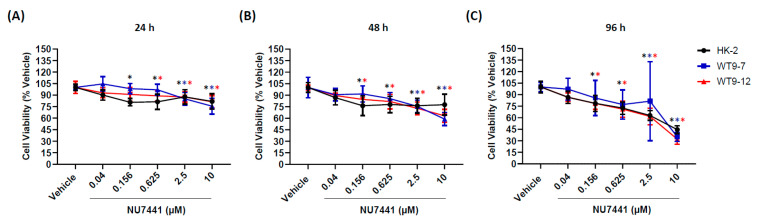
DNA-dependent protein kinase (DNA-PK) inhibition does not confer susceptibility to autosomal dominant polycystic kidney disease (ADPKD) cells. Percentage cell viability after (**A**) 24 h, (**B**) 48 h, and (**C**) 96 h of exposure to NU7441 treatment in normal kidney (HK-2) cells and ADPKD (WT9-7 and WT9-12) cell lines. Results are presented as mean ± standard deviation (*n* = 12 for each time point) after normalization to vehicle control. Significance was determined by Kruskal–Wallis followed by post hoc Dunn–Bonferroni test. * *p* < 0.01 versus vehicle control of the same cell line at the same time point (color of asterisk symbol represents line color of respective cell lines).

To evaluate whether the inhibition of DNA-PK caused synthetic lethality in human ADPKD cells, the anti-proliferative of NU7441 was compared to normal human kidney cells. As shown in Figure 4, the number of viable cells at various concentrations of NU7441 (0.04, 0.156, 0.625, 2.5, and 10 µM) in human ADPKD cell lines (WT9-7, WT9-12) was similar to a normal human kidney cell line (HK-2). Cell viability decreased with prolonged exposure and had less than 75% viability by 96 h at doses greater than 0.625 µM.

### 2.5. DNA-PK Inhibition Enhances the Anti-Proliferative Effects of Sirolimus in Both Human ADPKD and Normal Kidney Cells 

**Figure 5:**
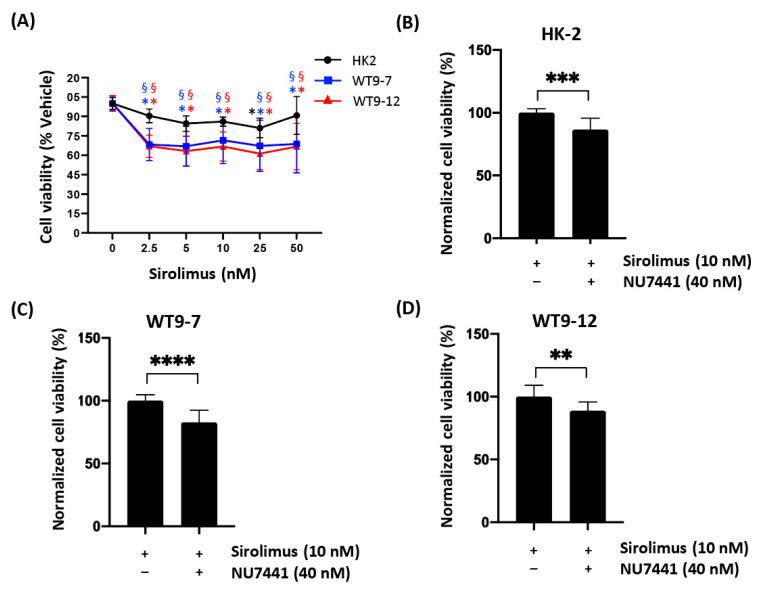
DNA-dependent protein kinase (DNA-PK) inhibition sensitizes autosomal dominant polycystic kidney disease (ADPKD) cells to mammalian target of rapamycin complex 1 (mTORC1) inhibition. Over 96 h, serial dilutions of sirolimus were tested as a single agent (**A**) in normal (HK-2) and ADPKD (WT9-7 and WT9-12) human cell lines. Cell viability (%) at all doses was analyzed compared to vehicle control of the same cell line. Results are presented as mean ± standard deviation (*n* = 12) after normalization to vehicle control. Significance was determined by Kruskal–Wallis followed by post hoc Dunn–Bonferroni test. One-way analysis of variance (ANOVA) and a post hoc Tukey honestly significant difference (HSD) test were carried out between cell lines. * *p* < 0.05 versus vehicle control of the same cell line; § *p* < 0.05 versus HK-2 cell line of the same dose and treatment. The color of symbols represent the line color of respective cell lines. In the same normal and ADPKD cell lines, sirolimus (10 nM) was tested in combination with low-dose NU7441 (40 nM) (**B**–**D**). Similarly, results are presented as mean cell viability (%) ± standard deviation (*n* = 12) after normalization to vehicle control and additional normalization to single therapy with 10nM sirolimus for comparison. Significance between treatments was tested by independent *t*-test for each cell line. ** *p* < 0.01, *** *p* < 0.001, **** *p* < 0.0001 between combined 10nM sirolimus and 40 nM NU7441 and 10 nM sirolimus treatment alone.

We next evaluated whether a non-toxic dose of NU7441 (0.04 µM; Figure 4) could enhance the anti-proliferative effects of TORC1 inhibition at sub-therapeutic doses (2.5 to 50 nM sirolimus) on ADPKD cells compared to normal kidney cells. As shown in Figure 5A, sirolimus alone, at the lowest dose (2.5 nM), reduced the number of viable cells by 31 ± 2% and 35 ± 3% in WT9-7 and WT9-12 cells, respectively (*p* < 0.05), and, interestingly, this level of suppression was similar at higher doses. In contrast, in HK-2 cells the number of viable cells was only reduced at 25nM of sirolimus (19% reduction in the number of viable cells; *p* < 0.05 compared to vehicle). There was a greater reduction in the number of viable cells in WT9-7/WT9-12 cells compared to HK-2 cells, suggesting that ADPKD cells are more sensitive to the anti-proliferative effects of sirolimus (Figure 5A).

To determine whether DNA-PK inhibition enhanced the anti-proliferative effects of TORC1 inhibition, we compared the effects of NU7441+sirolimus with sirolimus alone in the cell lines. As shown in Figure 5, NU7441+sirolimus further reduced the number of viable cells by 17 ± 10% (*p* < 0.05) and 11 ± 7% (*p* < 0.05) in WT9-7 and WT9-12 cells, respectively, compared to sirolimus alone (Figure 5C,D). Similarly, in HK-2 cells, the combined treatment reduced the number of viable cells by 13 ± 9% compared to sirolimus alone (*p* < 0.05).

## 3. Discussion

In this study, the hypothesis that DNA-PK expression is increased in human ADPKD and that inhibition of this DNA repair kinase reduces cyst growth in vitro was evaluated. The key findings were the following: (i) the expression of genes of all three subunits of the DNA-PK complex was increased in human ADPKD transcriptome, as well as localized to cyst lining epithelial cells in human ADPKD; (ii) long-term inhibition of the catalytic activity using specific small molecule inhibitor NU7441 in MDCK 3D cysts reduced MDCK growth by up to 52%; (iii) human ADPKD cells do not exhibit synthetic lethality to DNA-PK inhibition by NU7441; and (iv) finally, the increased sensitivity of human ADPKD cells to TORC1 inhibition was enhanced by combination treatment with NU7441.

On analysis of public transcriptome datasets from ADPKD patients compared to the normal renal cortex, we found that the catalytic subunit of DNA-PK, essential for its function as a kinase and in NHEJ, is increased in a manner that is positively correlated to increased cyst size. Interestingly, the expression of catalytic subunits in the nuclei by immunohistochemistry was sporadic and did not affect all cysts in human ADPKD. The discrepancy between these findings could be due to the differences in technique used to evaluate DNA-PK, as well as our immunohistochemistry being limited to only one protein epitope.

To further evaluate the potential function of DNA-PK in ADPKD, the effects of a specific inhibitor, NU7741, were examined. In vitro, NU7441 reduced cyst diameter in a dose-dependent manner at doses that did not reduce the number of viable cells. Using an MTT assay, we determined that although the reduction in cyst size at 2.5 µM could be due to a reduction in cell numbers, the slower cyst growth at 0.625 µM could not be explained by this mechanism, and alternate mechanisms, such as apoptosis or alterations in intra-cystic fluid accumulation, are possible. Consistent with the results of the present study, PIK-75 (a dual inhibitor of DNA-PK and phosphoinositide 3-kinase (PI3K)), reduced cyst growth in a murine IMCD3 forskolin-induced cyst model [24]. However, this was characterized by a low viable cell count and not further examined. In contrast, the same study tested NU7441 in the IMCD3 cysts but did not identify any change in cyst size in the dose range tested in the MDCK cyst model in the current study [24], suggesting possible species and/or disease-specific effects. Unraveling the additional mechanisms by which DNA-PK inhibition influences MDCK cyst growth warrants further investigation.

Contrary to the main hypothesis, we did not find evidence that DNA-PK inhibition induces synthetic lethality in human ADPKD cells, as the number of viable cells on exposure to NU7441 was similar to normal kidney cells. Previous literature indicated variability in the toxicity of NU7441, where normal human fibroblasts tolerated up to 1 µM [30], whereas treatment of normal renal HK-2 cells with NU7441 and NU7026 did not demonstrate toxicity at doses of up to 5µM [31]. In contrast, we found that concentrations above 0.156 µM affected both human ADPKD cell lines, WT9-7 and WT9-12, and normal renal HK-2 cells to a similar extent. These findings suggest that long-term use of NU7441 may have off-target toxicity to normal parenchyma if used continuously and may require a pulsed treatment approach. Further in vivo studies are needed to test this hypothesis.

Finally, we evaluated the effect of a combined treatment of sirolimus with NU7441 on the viability of human ADPKD and normal kidney cell lines. As NU7441 did not affect the number of viable cells at a concentration of 0.04 µM of NU7441 in any cell line, we postulated that this dose, combined with an equivalent sub-therapeutic dose of sirolimus, might be efficacious and potentially be a strategy to overcome the toxicity of using TORC1 inhibitors alone in human ADPKD [28,29]. Similar strategies have been harnessed for the treatment of various malignancies where DNA-PK inhibition renders malignant cells susceptible to chemo- and radio-therapies [32,33,34,35]. In addition, dual DNA-PK and TORC1 inhibition is under investigation in cancer but has not been evaluated as a potential therapy in ADPKD. Our results showed that low-dose NU7441 sensitized the anti-proliferative effects of sirolimus in human ADPKD cells, but the effect was modest. As these results suggest dual DNA-PK and TORC1 inhibition might reduce kidney cyst growth in vivo, further studies using a genetic ortholog of ADPKD would be an important next step.

In conclusion, the results of this study show, for the first time, that the expression of DNA-PK is increased in human ADPKD. However, our data indicate that monotherapy with DNA-PK inhibitors may not be suitable for long-term treatment of attenuating cyst growth in vivo, given that there is no evidence of the synthetic lethality of this pathway. On the other hand, it is possible that there may be an opportunity for combining DNA-PK inhibitors with TORC1 inhibitors to minimize the off-target and toxic effects of the latter through dose reduction. Further preclinical studies, testing the pharmacological inhibition in vivo, are warranted to fully evaluate this possibility and the potential of DNA-PK inhibition as a disease-modifying therapy in ADPKD.

## 4. Materials and Methods

### 4.1. Expression of DNA-PK in Human ADPKD Transcriptome

The gene expression values of proteins encoding DNA-PKcs, Ku 70, and Ku 80 (*PRKDC, XRCC5,* and *XRCC6,* respectively) were filtered from published data of DDR genes in ADPKD [11]. Briefly, the samples consisted of human ADPKD cysts of varying sizes (GSE7869; *n* = 18) [36] and normal kidney (GSE7869; *n* = 3, GSE9493; *n* = 10) [37,38]. Comparisons were performed between ADPKD and normal kidney, and between cyst sizes in ADPKD tissue (minimally cystic tissue (*n* = 5), small (<1 mL; *n* = 5), medium (10–25 mL; *n* = 5) and large (>50 mL; *n* = 3) cysts).

### 4.2. Immunohistochemistry for DNA-PK in Human ADPKD

Paraffin-embedded kidney tissue from ADPKD patients (*n* = 6) and non-cancerous portions of renal tissue (*n* = 2) were obtained from archival samples from a previously published study [11]. Written informed consent was provided by all patients, and the study was approved by the Human Research Ethics Committee at Westmead Hospital (HREC/09/WMEAD/305; SSA/12/WMEAD/327). Coronal sections of human end-stage and normal kidneys were incubated overnight in primary antibodies; either anti-p-DNA-PKcs (S2056) (1:400, ab18192; Abcam) or anti-DNA-PKcs total protein (1:500; ab32566; Abcam), followed by secondary antibody; either HRP-conjugated goat anti-rabbit (1:200; Sigma-Aldrich, St Louis, MO, USA) or biotinylated goat anti-rabbit (1:200; Sigma-Aldrich, St Louis, MO, USA). All antibodies were diluted in DaVinci Green diluent (PD900; BioCare Medical, Concord, CA, USA). For biotinylated samples, the signal was amplified using Vectastain R.T.U. ABC reagent (Vector Laboratories, Burlingame, CA, USA) for 20 min. Slides were stained with chromogen 3,3’-Diaminobenzidine (DAB) solution (Dako Agilent Technologies, Santa Clara, CA, USA) and counterstained with methyl green (Sigma-Aldrich, St Louis, MO, USA). Digital image acquisition was performed using a NanoZoomer slide scanner (v1, Hamamatsu Photonics, Hamamatsu City, Shizuoka, Japan) and analyzed using Aperio ImageScope (v11.2.0.780, Leica Biosystems, Wetzlar, Germany).

### 4.3. Cell Lines

Immortalized cell lines from healthy human kidney (HK-2; CRL-2190, Lot no. 61218770), human ADPKD (WT9-7; CRL-2830, Lot no. 58737172 and WT9-12; CRL-2833, Lot no. 60336584) [39], and Madin-Darby canine kidney (MDCK) epithelial cells were obtained from the American Type Culture Collection (ATCC, Manassas, VA, USA), as previously described [11]. Short tandem repeat (STR) DNA profiling was performed for authentication of human cell lines. WT9-7 and WT9-12 cells were cultured in Dulbecco’s minimum essential Mmedium (DMEM)(GIBCO, Thermo Fisher Scientific, Waltham, MA, USA), and HK-2 and MDCK cells were cultured in a 1:1 ratio of DMEM and Ham’s F12 (GIBCO, Thermo Fisher Scientific, Waltham, MA, USA). The media were supplemented with 10% fetal bovine serum (FBS) and the cultures were maintained at 37 °C, 5% CO_2_.

### 4.4. Effect of NU7441 Treatment on Viability of ADPKD Cells

Cell viability was assayed using an MTT (3-(4,5-dimethylthiazol-2-yl)-2,5-diphenyltetrazolium bromide) assay, as per manufacturer protocol (11465007001, Roche Diagnostics, Mannheim, Germany). Briefly, MDCK, HK-2, WT9-7, and WT9-12 cells were seeded at 2 × 10^3^ cells/well in 100 µL media in a 96-well plate and treated with NU7441, either alone at varying concentrations [31,40,41] or combined with sirolimus at concentrations below therapeutic blood levels (2.5–50 nM) [42,43,44,45]. NU7441 is a highly selective and potent ATP-competitive DNA-PK inhibitor, with an IC_50_ of 14nM [41]. NU7441 was added at four-fold serial dilutions from 0 to10 µM, with the 0µM vehicle control containing dimethyl sulfoxide (DMSO) at a volume equal to the highest dose. Cells were incubated with the drug for 24, 48, and 96 h. To test sirolimus +/− NU7441, sirolimus doses were tested, including, and below, therapeutic blood plasma levels (2.5–50 nM) of the drug in combination with a non-toxic dose of NU7441 (40 nM) (media were changed every two days). The assay (Roche Diagnostics, Mannheim, Germany) was performed according to the manufacturer’s specifications. Following drug treatment, MTT solution (0.5 mg/mL) was added to each well and incubated for 4 h to allow the development of formazan crystals by normal metabolic activity. Solubilization solution was then added followed by overnight incubation. Absorbance was measured the following day at 570 nm (720 nm reference). Percentage viability was calculated for each sample as 100 * (absorbance/average absorbance of vehicle control). Measurements were taken for *n* = 4 replicates per treatment group over three repeat experiments (total *n* = 12) at each time point.

### 4.5. Effect of NU7441 Treatment on Three-Dimensional (3D) MDCK Cyst Model

Three-dimensional cysts of MDCK cells was grown, as previously described [46]. Briefly, MDCK cells in collagen were incubated in the continuous presence of 10 µM forskolin to induce cyst growth and treated with either vehicle (DMSO), 0.625 µM, 2.5 µM, or 10 µM of DNA-PK inhibitors; NU7441 (IC_50_ of 14 nM), LTURM34 (IC_50_ of 34 nM), and NU7026 (IC_50_ of 230 nM), or 0.05 µM sirolimus (positive control). All drugs were obtained from Selleck Chemicals (Houston, TX, USA). Media with treatment were refreshed every two days, and images were obtained at days 6 and 12. Cyst diameter was determined for 60 randomly selected cysts per treatment (20 cysts/well × triplicates) at each time point using Image J (v1.52a, U.S. National Institutes of Health, Bethesda, MD, USA). Experiments were repeated thrice for a total of *n* = 180 for vehicle and 0.625µM, and twice for a total of *n* = 120 for 2.5 µM NU7441 at each time point.

### 4.6. Statistical Analyses

For statistical analysis of gene expression data, fold changes were transformed to a linear scale by log 2 transformation. All data were analyzed using the JMP^®^ Pro statistical software package (v14.2.0, SAS Institute, Cary, NC, USA) and graphed using GraphPad Prism (v8.2.1, San Diego, CA, USA). A Shapiro–Wilks test was performed to determine the normality of distribution of data. Variance of data was assessed by Bartlett’s test for normally distributed data and Levene test for data that did not follow a normal distribution. An independent T-test was used to compare two normally distributed datasets. For multiple groups, Kruskal–Wallis and Dunn–Bonferroni post hoc analysis or one-way analysis of variance (ANOVA) and post hoc analysis by Tukey Kramer honestly significant difference (HSD) tests were carried out, depending on the distribution of data. *p*-values less than 0.05 was defined as statistically significant. For gene expression data, *p*-values were adjusted for false discovery rate (FDR) and presented as *q*-values.

## Figures and Tables

**Table 1:** Gene expression of DNA-PK subunits in human ADPKD compared to normal kidney (*n* = 13), by cyst size. Data are presented as the fold-change ±SD.

Gene	Tissue	Fold Change *	
		Average	95% Confidence Interval
*PRKDC*	Normal (*n* = 13)	1.00	(0.80, 1.25)
	ADPKD (*n* = 18)	2.12 ^a^	(1.78, 2.53)
	Minimally cystic (*n* = 5)	1.41 ^c^	(1.13, 1.76)
	Small cysts (<1 mL; *n* = 5)	2.04 ^b^	(1.41, 2.95)
	Medium cysts (10–25 mL; *n* = 5)	2.61 ^a^	(1.17, 3.05)
	Large cysts (>50 mL; *n* =3)	3.18 ^a^	(1.41, 4.47)
*XRCC5*	Normal (*n* = 13)	1.00	(0.84, 1.19)
	ADPKD (*n* = 18)	1.79 ^b^	(1.68, 1.90)
	Minimally cystic (*n* = 5)	1.72 ^b^	(1.22, 2.10)
	Small cysts (<1 mL; *n* = 5)	1.83 ^a^	(1.51, 2.19)
	Medium cysts (10–25 mL; *n* = 5)	1.71 ^b^	(1.51, 1.93)
	Large cysts (>50 mL; *n* = 3)	1.97 ^a^	(1.75, 2.22)
*XRCC6*	Normal (*n* = 13)	1.00	(0.89, 1.12)
	ADPKD (*n* = 18)	1.65 ^a^	(1.53, 1.78)
	Minimally cystic (*n* = 5)	1.45 ^c^	(1.15, 1.82)
	Small cysts (<1 mL; *n* = 5)	1.63 ^a^	(1.48, 1.80)
	Medium cysts (10–25 mL; *n* = 5)	1.81 ^a^	(1.53, 2.15)
	Large cysts (>50 mL; *n* = 3)	1.77 ^a^	(1.58, 1.98)

* Fold changes are relative to the average expression of normal tissue (*n* = 13). Mean and 95% confidence intervals were calculated using log 2 transformed fold change (Log2FC). The *p* values of all the presented data are <0.01. *p*-values adjusted for false discovery rate (FDR) are presented (*q*); ^a^ *q* < 0.001; ^b^ *q* < 0.01; ^c^ *q* < 0.05, when compared to normal tissue.

## Data Availability

All data generated and reported in this study have been presented as tables and/or figures within this article.

## References

[1] Reeders S.T., Breuning M.H., Davies K.E., Nicholls R.D., Jarman A.P., Higgs D.R., Pearson P.L., Weatherall D.J. (1985). A highly polymorphic DNA marker linked to adult polycystic kidney disease on chromosome 16. Nature.

[2] Harris P.C., Ward C.J., Peral B., Hughes J. (1995). Polycystic kidney disease. 1: Identification and analysis of the primary defect. J. Am. Soc. Nephrol..

[3] Mochizuki T., Wu G., Hayashi T., Xenophontos S.L., Veldhuisen B., Saris J.J., Reynolds D.M., Cai Y., Gabow P.A., Pierides A. (1996). PKD2, a Gene for Polycystic Kidney Disease That Encodes an Integral Membrane Protein. Science.

[4] Rossetti S., Kubly V.J., Consugar M.B., Hopp K., Roy S., Horsley S.W., Chauveau D., Rees L., Barratt T.M., Hoff W.G.V. (2009). Incompletely penetrant PKD1 alleles suggest a role for gene dosage in cyst initiation in polycystic kidney disease. Kidney Int..

[5] Hopp K., Ward C.J., Hommerding C.J., Nasr S.H., Tuan H.-F., Gainullin V.G., Rossetti S., Torres V.E., Harris P.C. (2012). Functional polycystin-1 dosage governs autosomal dominant polycystic kidney disease severity. J. Clin. Investig..

[6] Leeuwen I.S.L.-V., Dauwerse J.G., Baelde H.J., Leonhard W.N., van de Wal A., Ward C.J., Verbeek S., DeRuiter M.C., Breuning M.H., de Heer E. (2004). Lowering of Pkd1 expression is sufficient to cause polycystic kidney disease. Hum. Mol. Genet..

[7] Tan A.Y., Zhang T., Michaeel A., Blumenfeld J., Liu G., Zhang W., Zhang Z., Zhu Y., Rennert L., Martin C. (2018). Somatic Mutations in Renal Cyst Epithelium in Autosomal Dominant Polycystic Kidney Disease. J. Am. Soc. Nephrol..

[8] Koptides M., Hadjimichael C., Koupepidou P., Pierides A., Deltas C.C. (1999). Germinal and somatic mutations in the PKD2 gene of renal cysts in autosomal dominant polycystic kidney disease. Hum. Mol. Genet..

[9] Conduit S.E., Davies E.M., Ooms L.M., Gurung R., McGrath M., Hakim S., Cottle D.L., Smyth I., Dyson J.M., Mitchell C.A. (2020). AKT signaling promotes DNA damage accumulation and proliferation in polycystic kidney disease. Hum. Mol. Genet..

[10] Li M., Qin S., Wang L., Zhou J. (2013). Genomic instability in patients with autosomal-dominant polycystic kidney disease. J. Int. Med Res..

[11] Zhang J.Q., Saravanabavan S., Chandra A.N., Munt A., Wong A.T., Harris P.C., Harris D.C., McKenzie P., Wang Y., Rangan G.K. (2021). Up-Regulation of DNA Damage Response Signaling in Autosomal Dominant Polycystic Kidney Disease. Am. J. Pathol..

[12] Ta M.H., Schwensen K.G., Liuwantara D., Huso D.L., Watnick T., Rangan G.K. (2016). Constitutive renal Rel/nuclear factor-κB expression in Lewis polycystic kidney disease rats. World J. Nephrol..

[13] Zhang J.Q.J., Saravanabavan S., Munt A., Wong A.T.Y., Harris D.C., Harris P.C., Wang Y., Rangan G.K. (2019). The role of DNA damage as a therapeutic target in autosomal dominant polycystic kidney disease. Expert Rev. Mol. Med..

[14] Ciccia A., Elledge S.J. (2010). The DNA Damage Response: Making It Safe to Play with Knives. Mol. Cell.

[15] Shrivastav M., De Haro L.P., Nickoloff J.A. (2008). Regulation of DNA double-strand break repair pathway choice. Cell Res..

[16] Chen X., Xu X., Chen Y., Cheung J.C., Wang H., Jiang J., de Val N., Fox T., Gellert M., Yang W. (2021). Structure of an activated DNA-PK and its implications for NHEJ. Mol. Cell.

[17] Wang C.-Y., Huang E.Y.-H., Huang S.-C., Chung B.-C. (2015). DNA-PK/Chk2 induces centrosome amplification during prolonged replication stress. Oncogene.

[18] Chen T.-Y., Huang B.-M., Tang T.K., Chao Y.-Y., Xiao X.-Y., Lee P.-R., Yang L.-Y., Wang C.-Y. (2021). Genotoxic stress-activated DNA-PK-p53 cascade and autophagy cooperatively induce ciliogenesis to maintain the DNA damage response. Cell Death Differ..

[19] Park S.-J., Gavrilova O., Brown A.L., Soto J.A., Bremner S., Kim J., Xu X., Yang S., Um J.-H., Koch L.G. (2017). DNA-PK Promotes the Mitochondrial, Metabolic, and Physical Decline that Occurs During Aging. Cell Metab..

[20] Dionne L.K., Shim K., Hoshi M., Cheng T., Wang J., Marthiens V., Knoten A., Basto R., Jain S., Mahjoub M.R. (2018). Centrosome amplification disrupts renal development and causes cystogenesis. J. Cell Biol..

[21] Padovano V., Podrini C., Boletta A., Caplan M.J. (2018). Metabolism and mitochondria in polycystic kidney disease research and therapy. Nat. Rev. Nephrol..

[22] Nowak K.L., Hopp K. (2020). Metabolic Reprogramming in Autosomal Dominant Polycystic Kidney Disease: Evidence and Therapeutic Potential. Clin. J. Am. Soc. Nephrol..

[23] Battini L., Macip S., Fedorova E., Dikman S., Somlo S., Montagna C., Gusella G.L. (2008). Loss of polycystin-1 causes centrosome amplification and genomic instability. Hum. Mol. Genet..

[24] Booij T., Bange H., Leonhard W., Yan K., Fokkelman M., Kunnen S., Dauwerse J.G., Qin Y., Van De Water B., van Westen G. (2017). High-Throughput Phenotypic Screening of Kinase Inhibitors to Identify Drug Targets for Polycystic Kidney Disease. SLAS Discov. Adv. Life Sci. R&D.

[25] Tao Y., Kim J., Schrier R.W., Edelstein C.L. (2005). Rapamycin Markedly Slows Disease Progression in a Rat Model of Polycystic Kidney Disease. J. Am. Soc. Nephrol..

[26] Shillingford J.M., Murcia N.S., Larson C.H., Low S.H., Hedgepeth R., Brown N., Flask C.A., Novick A.C., Goldfarb D.A., Kramer-Zucker A. (2006). The mTOR pathway is regulated by polycystin-1, and its inhibition reverses renal cystogenesis in polycystic kidney disease. Proc. Natl. Acad. Sci. USA.

[27] Wu M., Wahl P.R., Le Hir M., Wäckerle-Men Y., Wüthrich R.P., Serra A.L. (2007). Everolimus Retards Cyst Growth and Preserves Kidney Function in a Rodent Model for Polycystic Kidney Disease. Kidney Blood Press. Res..

[28] Serra A.L., Poster D., Kistler A.D., Krauer F., Raina S., Young J., Rentsch K.M., Spanaus K.S., Senn O., Kristanto P. (2010). Sirolimus and kidney growth in autosomal dominant polycystic kidney disease. N. Engl. J. Med..

[29] Walz G., Budde K., Mannaa M., Nürnberger J., Wanner C., Sommerer C., Kunzendorf U., Banas B., Hörl W.H., Obermüller N. (2010). Everolimus in Patients with Autosomal Dominant Polycystic Kidney Disease. N. Engl. J. Med..

[30] Song X., Di Giovanni V., He N., Wang K., Ingram A., Rosenblum N.D., Pei Y. (2009). Systems biology of autosomal dominant polycystic kidney disease (ADPKD): Computational identification of gene ex-pression pathways and integrated regulatory networks. Hum. Mol. Genet..

[31] Rödder S., Scherer A., Raulf F., Berthier C.C., Hertig A., Couzi L., Durrbach A., Rondeau E., Marti H.-P. (2009). Renal Allografts with IF/TA Display Distinct Expression Profiles of Metzincins and Related Genes. Am. J. Transplant..

[32] Saint-Mezard P., Berthier C.C., Zhang H., Hertig A., Kaiser S., Schumacher M., Wieczorek G., Bigaud M., Kehren J., Rondeau E. (2009). Analysis of independent microarray datasets of renal biopsies identifies a robust transcript signature of acute allograft rejection. Transpl. Int..

[33] Loghman-Adham M., Nauli S.M., Soto C.E., Kariuki B., Zhou J. (2003). Immortalized epithelial cells from human autosomal dominant polycystic kidney cysts. Am. J. Physiol. Renal Physiol..

[34] Zheng B., Mao J.-H., Li X.-Q., Qian L., Zhu H., Gu D.-H., Pan X.-D. (2016). Over-expression of DNA-PKcs in renal cell carcinoma regulates mTORC2 activation, HIF-2α expression and cell proliferation. Sci. Rep..

[35] Jan Y.-H., Heck D.E., Laskin D.L., Laskin J.D. (2019). Sulfur Mustard Analog Mechlorethamine (Bis(2-chloroethyl)methylamine) Modulates Cell Cycle Progression via the DNA Damage Response in Human Lung Epithelial A549 Cells. Chem. Res. Toxicol..

[36] Leahy J.J., Golding B.T., Griffin R.J., Hardcastle I.R., Richardson C., Rigoreau L., Smith G.C. (2004). Identification of a highly potent and selective DNA-dependent protein kinase (DNA-PK) inhibitor (NU7441) by screening of chromenone libraries. Bioorg. Med. Chem. Lett..

[37] Meier-Kriesche H.-U., Kaplan B. (2000). Toxicity and efficacy of sirolimus: Relationship to whole-blood concentrations. Clin. Ther..

[38] Novalic Z., Van Der Wal A.M., Leonhard W., Koehl G., Breuning M.H., Geissler E.K., De Heer E., Peters D.J. (2012). Dose-Dependent Effects of Sirolimus on mTOR Signaling and Polycystic Kidney Disease. J. Am. Soc. Nephrol..

[39] Choo S., Chowbay B., Ng Q., Thng C., Lim C., Hartono S., Koh T., Huynh H., Poon D., Ang M. (2013). A Phase 1 dose-finding and pharmacodynamic study of rapamycin in combination with bevacizumab in patients with unresectable hepatocellular carcinoma. Eur. J. Cancer.

[40] Holditch S.J., Brown C.N., Atwood D.J., Lombardi A.M., Nguyen K.N., Toll H.W., Hopp K., Edelstein C.L. (2019). A study of sirolimus and mTOR kinase inhibitor in a hypomorphic Pkd1 mouse model of autosomal dominant polycystic kidney disease. Am. J. Physiol. Renal Physiol..

[41] Zhang J.Q.J., Saravanabavan S., Rangan G.K. (2021). Effect of Reducing Ataxia-Telangiectasia Mutated (ATM) in Experimental Autosomal Dominant Polycystic Kidney Disease. Cells.

[42] Sunada S., Kanai H., Lee Y., Yasuda T., Hirakawa H., Liu C., Fujimori A., Uesaka M., Okayasu R. (2016). Nontoxic concentration of DNA—PK inhibitor NU7441 radio-sensitizes lung tumor cells with little effect on double strand break repair. Cancer Sci..

[43] Timme C.R., Rath B.H., O’neill J.W., Camphausen K., Tofilon P.J. (2018). The DNA-PK Inhibitor VX-984 Enhances the Radiosensitivity of Glioblastoma Cells Grown In Vitro and as Orthotopic Xenografts. Mol. Cancer Ther..

[44] Wise H.C., Iyer G.V., Moore K., Temkin S.M., Gordon S., Aghajanian C., Grisham R.N. (2019). Activity of M3814, an Oral DNA-PK Inhibitor, In Combination with Topoisomerase II Inhibitors in Ovarian Cancer Models. Sci. Rep..

[45] Fok J.H.L., Ramos-Montoya A., Vazquez-Chantada M., Wijnhoven P.W.G., Follia V., James N., Farrington P.M., Karmokar A., Willis S.E., Cairns J. (2019). AZD7648 is a potent and selective DNA-PK inhibitor that enhances radiation, chemotherapy and olaparib activity. Nat. Commun..

[46] Willoughby C.E., Jiang Y., Thomas H.D., Willmore E., Kyle S., Wittner A., Phillips N., Zhao Y., Tudhope S.J., Prendergast L. (2020). Selective DNA-PKcs inhibition extends the therapeutic index of localized radiotherapy and chemotherapy. J. Clin. Investig..

